# Bovine Alphaherpesvirus 2 infections in Bavaria: an analysis of the current situation - several years after eradicating Bovine Alphaherpesvirus 1

**DOI:** 10.1186/s12917-020-02310-w

**Published:** 2020-05-24

**Authors:** Stefanie Singer, Bernd Hoffmann, Angela Hafner-Marx, Jürgen Christian, Friederike Forster, Katharina Schneider, Gabriela Knubben-Schweizer, Antonie Neubauer-Juric

**Affiliations:** 1 Bavarian Health and Food Safety Authority, Veterinärstrasse 2, 85764, Oberschleißheim, Germany; 2 Institute of Diagnostic Virology, Friedrich-Loeffler-Institute, Insel Riems, Germany; 3 Bavarian Health and Food Safety Authority, Erlangen, Germany; 4 Clinic for Ruminants with Ambulatory and Herd Health Services, Ludwig-Maximilians-Universität München, Oberschleißheim, Germany

**Keywords:** Bovine Alphaherpesvirus 2, Bovine Alphaherpesvirus 1, BoHV-2, BoHV-1, Seroprevalence, Real time PCR, Bovine herpes mammillitis, Bavaria, Germany

## Abstract

**Background:**

Bavaria, a large federal state in Germany, has been declared free from infections with Bovine Alphaherpesvirus 1 (BoHV-1) in 2011. To maintain this status the cattle population is monitored for antibodies against BoHV-1 regularly. Several years ago, infrequent but recurrent problems in this sero-surveillance were statistically put into correlation with the presence of antibodies against Bovine Alphaherpesvirus 2 (BoHV-2). In Europe, BoHV-2 is primarily known as the agent causing bovine herpes mammillitis. However, very little information about BoHV-2 infections in Bavaria is available so far. Therefore, the aims of this study were to determine BoHV-2 seroprevalences and to detect virus genomes in potential clinical samples.

**Results:**

6801 blood sera of healthy cattle from all over Bavaria were tested for antibodies against BoHV-2, revealing an overall seroprevalence of 5.51%. Interestingly, seroprevalences markedly varied between the North and the South of Bavaria, namely from 0.42 to 11.17%. Concurrently, the previously reported relation between the epidemiologically inexplicable sero-reactivities in BoHV-1 ELISAs and the presence of BoHV-2 infections were statistically corroborated in this study. To detect BoHV-2 genomes a fast and sensitive real time PCR was established. Using a multiple PCR strategy, tissue samples from skin lesions at relevant localizations, corresponding lymph nodes, and trigeminal ganglia from 111 animals, as well as nasal swabs from 918 bovines with respiratory symptoms were tested. However, BoHV-2 genomes were not detected in any of these samples.

**Conclusions:**

BoHV-2 antibodies were found in samples from bovines all over Bavaria, albeit with an explicit South-North-divide. BoHV-2 genomes, however, could not be detected in any of the analyzed samples, indicating that acute clinical cases as well as obvious virus reactivation are relatively rare. Consequently, the future spread of BoHV-2 infections throughout Bavaria, particularly, after eradicating BoHV-1, has to be further monitored.

## Background

*Bovine Alphaherpesvirus 2* (BoHV-2) has been assigned to the genus *Simplexvirus* of the subfamily *Alphaherpesvirinae* in the family *Herpesviridae* [1]. In Europe the virus is primarily known as the causative agent of bovine herpes mammillitis (BHM), an ulcerative disease which typically affects the skin of the udder and the teats of lactating or recently dried-off cows [2, 3]. Lesions at the muzzle or the oral mucosa of calves have also been described [4, 5]. Intensity and duration of clinical symptoms differ between individuals and can range from subclinical to particularly severe or even atypical courses [2, 3, 6, 7]. The virus is present in large amounts in vesicular fluids and is in vitro isolable during early stages of the disease [8].

In addition to transmission by direct contact, indirect, mechanical alternatives, such as the process of milking or via biting flies, are discussed. At any rate, the intact skin represents an effective barrier to local infection implicating a role for pre-existing skin lesions [9]. In experimental settings, reactivation after latency and virus shedding via skin lesions but also via the nasal, the oral, or even via the vaginal mucosa have been demonstrated [8, 10–13]. Presumably latent BoHV-2 genomes have been detected in trigeminal ganglia of healthy cattle [14] or in trigeminal ganglia, tonsils, and regional lymph nodes of experimentally infected sheep [15]. However, the epidemiological relevance of these findings needs to be further assessed.

Case reports and serological studies show that BoHV-2 infections occur worldwide in domestic cattle and to some extend in wild ruminant populations (for example [16–22]). On the one hand, the wide variation of clinical signs and intra-herd prevalences, even in acute disease outbreaks, is demonstrated [17, 23]. On the other hand, studies focusing on serological data indicate relatively high prevalences of BoHV-2 antibodies in various cattle populations, although clinical symptoms are rarely observed and virologic confirmation is lacking [19, 20, 24]. These early serological studies, with data from the 1960s to 2000, mostly indicate antibody prevalences within the respective sample populations of about 6% to nearly 40% [17, 19, 20, 24, 25]. A relation to age as well as regional differences were noted. For example, BoHV-2 seroprevalences estimated from samples taken at regional slaughterhouses in Switzerland ranged from 1% in Western Switzerland to 19% in the canton of Ticino [24].

In Germany, particularly in Bavaria, BoHV-2 sero-reactivity came into focus quite recently when the presence of BoHV-2 antibodies within herds was reported to be related to otherwise inexplicable reactions in the sero-surveillance of *Bovine Alphaherpesvirus 1* (BoHV-1; order *Herpesvirales*, family *Herpesviridae*, subfamily *Alphaherpesvirinae*, genus *Varicellovirus*) infections [26].

Bavaria, a federal state of southern Germany, is officially listed as free from infections with BoHV-1, according to article 10 of directive 64/432/EEC since 2011 [27]. To monitor BoHV-1 freedom, bovines have to be tested regularly for the absence of BoHV-1 antibodies, following national regulations. This is achieved either by analyzing bulk milk or, if necessary, blood samples. In doing so, epidemiologically non-feasible BoHV-1 antibody reactions are repeatedly detected, albeit at low ratios. Unexplainably, this phenomenon is observed especially in the southwest region of Bavaria, in Swabia [26]. A significant correlation between these epidemiologically non-feasible reactions in several BoHV-1 test systems and the BoHV-2 seroprevalence within a given herd was shown [26].

Looking at farms from Swabia, chosen for the occurrence of problematic, “non-negative” results in the BoHV-1 surveillance, BoHV-2 specific antibodies were detected in 35.4% of the samples, whereas for unproblematic Swabian farms a BoHV-2 seroprevalence of only 12.5% was noted [26]. Moreover, BoHV-2 antibodies were not found in samples stemming from North-Eastern regions of Bavaria, which leads to the assumption that the frequency of BoHV-2 infections differs regionally.

Similar observations were made for South Tirol in Northern Italy and parts of Austria (unpublished observations by Tavella, A. (South Tyrol, 2016) and Schiefer, P. (Austria, 2016)). Nevertheless, there is no direct cross reactivity between BoHV-1 and BoHV-2 specific antisera in the respective diagnostic systems [26, 28, 29]. Furthermore, BoHV-2 is clearly closer related, genetically as well as antigenetically, to the *Human alphaherpesviruses 1* and *2* (Herpes Simplex Virus 1 and 2, order *Herpesvirales*, family *Herpesviridae*, subfamily *Alphaherpesvirinae*, genus *Simplexvirus*) than to BoHV-1 [28, 30, 31]. A fact that is reflected by the assignation of the former viruses to the genus *Simplexvirus*, whereas the latter belongs into the genus *Varicellovirus* [1]. However, epidemiologic and virologic, but also sequence information on the BoHV-2 genome, is still sparse [30, 31]. Interestingly though, within an experimental setting it was demonstrated that reactivation of BoHV-1 interfered with that of BoHV-2 [32].

Considering the information available, it was hypothesized that currently BoHV-2 infections might mainly occur in the southwest of Bavaria and that the overall prevalence might be relatively low. Furthermore, it was assumed that in coming years the spread of BoHV-2 infections and – if connected - resulting problems in the BoHV-1 serosurveillance might gain in importance in regions where BoHV-1 was successfully eradicated. Therefore, the main aim of this study was to gather basic information necessary about BoHV-2 infections in the Bavarian cattle population. On the one hand, BoHV-2 seroprevalences had to be determined for Bavarian cattle. On the other hand, to identify current BoHV-2 infections in Bavaria by testing a large number of samples, a fast and sensitive real time PCR protocol had to be established. Finally, the putative correlation between reactions in BoHV-2 and BoHV-1 serological test systems had to be assessed for all regions of Bavaria more than six years after successfully eradicating BoHV-1.

## Results

### Analyses of bovine sera for cross neutralization against BoHV-1 and BoHV-2

In a first step, to exclude a direct cross neutralization between current BoHV-2 and BoHV-1 field antibodies, 37 known BoHV-2 antibody positive and 27 known BoHV-1 antibody positive sera were analyzed in parallel in BoHV-2 and BoHV-1 specific serum neutralization tests (SNT). BoHV-2 antibody positive sera specifically neutralized to titers of 11 to ≥90 in the BoHV-2 SNT and BoHV-1 sera neutralized to titers of 6 to ≥90 in the BoHV-1 specific test. No cross-neutralization occurred. Therefore, it was concluded that the respective SNTs allow for specific detection and differentiation of BoHV-1 and BoHV-2 antibodies.

### Comparative analyses between BoHV-2 specific ELISA and serum neutralization test

**Fig. 1:**
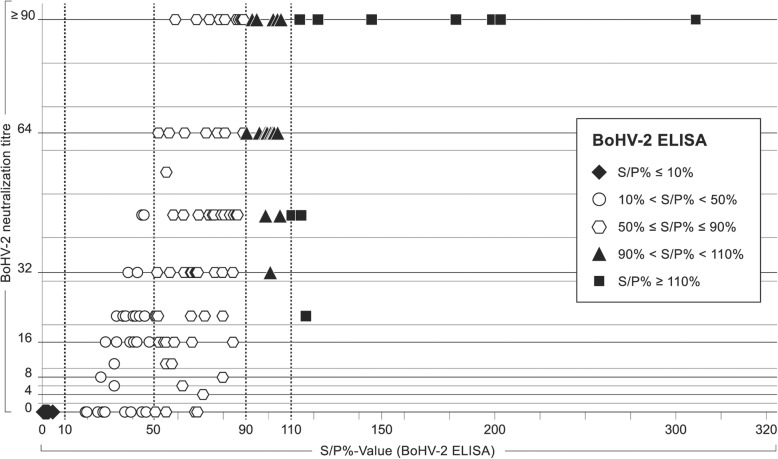
Correlation between BoHV-2 ELISA results and titers of neutralizing antibodies. Each symbol represents the results of one sample in both tests. The data for 128 samples are shown. ELISA results are indicated as S/P%-values. In case of duplicate analyses the mean S/P%-value per sample is given. Sera with ELISA values ≤90% are classified as negative by the manufacturer and are depicted as diamonds (♦) for S/P%-values ≤10%, circles (◯) for S/P%-values between 10 and 50%, or hexagons (⬡) for S/*P* values between 50 and 90% . Triangles (▲) indicate results classified as doubtful by ELISA (90% < S/P% < 110%). ELISA-positive sera are indicated by rectangles (■)

Of 6801 sera tested for seroprevalence calculations by BoHV-2 ELISA (ID Screen® BHV-2 Indirect; ID VET) (see below), the majority could be categorized either as clearly negative (6234 samples; S/P% ≤ 10%) or positive (298 samples; S/P% ≥ 110%). Further, the results of 20 sera lied within the given, doubtful range (90% < S/P% < 110%). Another 249 sera reacted within a range of 10% ≤ S/P% ≤ 90%. To assure a clear classification of these “in between” results a subset of 128 sera covering all possible ELISA reactions was chosen for comparative analysis in the BoHV-2 specific SNT. The results are summarized in Fig. 1.

BoHV-2 ELISA positive samples (*n* = 10) neutralized to titers of 22 to ≥90. Within this range, high ELISA-values (S/P% ≥ 150%) corresponded to high SNT titers (≥90). All eight samples with clearly negative ELISA values (S/P% ≤ 10%) showed no neutralizing activity at all, whereas all sera reacting doubtful (90% < S/P% < 110%; *n* = 20) neutralized to titers of 32 to ≥90. The range of 10% ≤ S/P% ≤ 90% was subdivided further into two categories. On the one hand, within the range of 50% ≤ S/P% ≤ 90%, hereafter called “increased”, 57 of 61 tested samples still generated titers of 4 to ≥90 while only four did not neutralize BoHV-2 infectivity. On the other hand, within the lower range of 10% < S/P% < 50%, already 9 of the 29 samples did not contain neutralizing antibodies and only 20 samples reacted to relatively low titers, namely between 4 and 45.

Taken together, in all ranges above 10% (S/P%) sera with low level but specific antibodies to BoHV-2 might be underestimated. As 93.4% of the samples within the increased range but only 69% of the sera with reactivities between 10 and 50% neutralized BoHV-2 antigen in SNT, a final cut-off value of 50% was chosen for the analyses in this study (Fig. 1). To avoid false positive results, sera with S/P%-values greater or equal 50 to 110% (“doubtful” or “increased”), were consequently retested for neutralizing antibodies and then finally rated according to the SNT results.

### Prevalences of BoHV-2 specific antibodies vary regionally

**Fig. 2:**
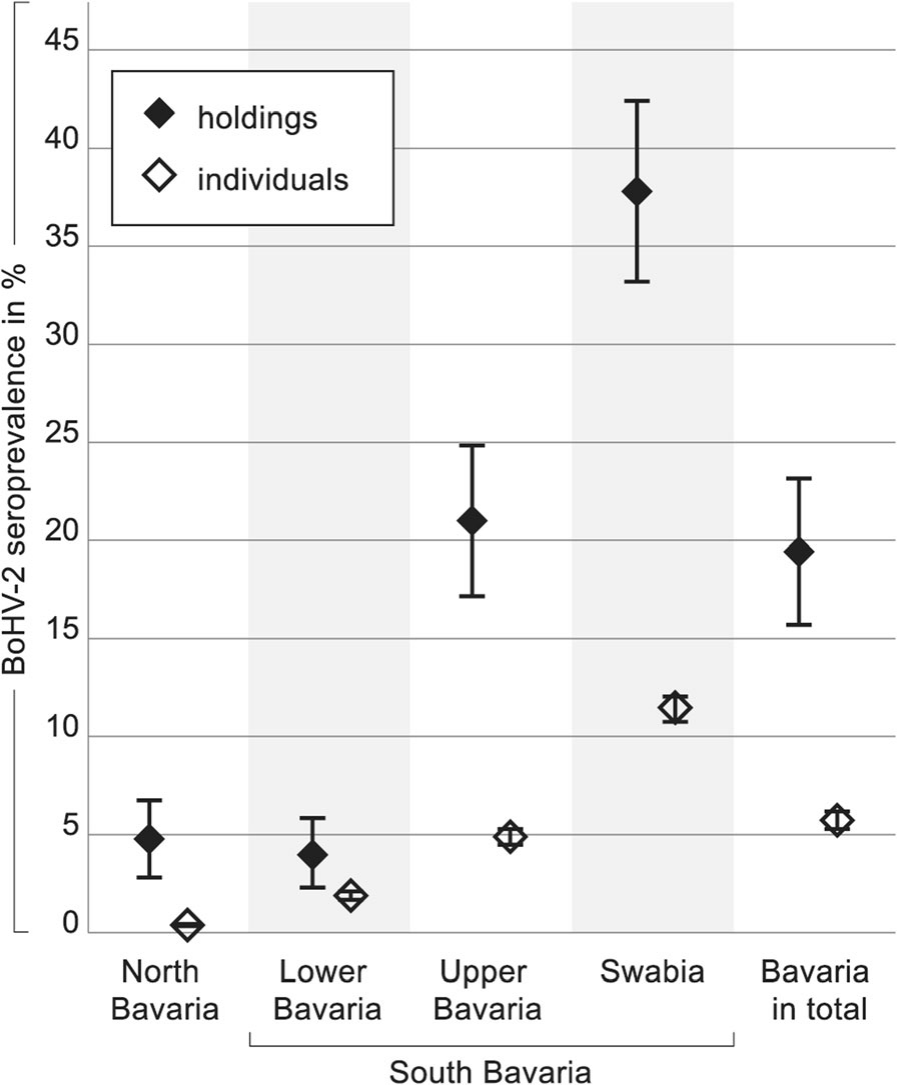
BoHV-2 seroprevalences in Bavaria divided into administrative regions. Seroprevalences calculated for 406 holdings are shown as black diamonds (♦), and those calculated for 6801 individual animals, irrespective of the correspondent farm, as open diamonds (◇). A holding is rated as “BoHV-2 seropositive” if at least one seropositive bovine animal is kept. Confidence intervals covering the true seroprevalence with a certainty of 95% are given

To estimate and to compare seroprevalences in Bavaria, all results were analyzed on the level of individual seropositive animals, as well as on the level of farms keeping at least one seropositive animal (= BoHV-2 seropositive farms; Fig. 2). Interestingly, 19.21% of all farms analyzed in Bavaria were categorized as BoHV-2 seropositive (78 of 406; CI 15.38–23.04%). However, looking at the regional distribution, marked differences became evident. Whereas in 4.76% of farms in the combined regions of North Bavaria (5 of 105; CI 2.69–6.83%), or 3.85% of farms in Lower Bavaria (3 of 78; CI 1.98–5.72%) at least one BoHV-2 seropositive bovine was found, this happened to be the circumstance for 20.73% of farms in Upper Bavaria (17 of 82; CI 16.79–24.67%), and even for 37.59% of farms in Swabia (53 of 141; CI 32.88–42.30%).

Regarding the individual animal level the BoHV-2 seroprevalences were clearly lower, namely 0.42% (8 of 1916; CI 0.26–0.57%) in North Bavaria, 1.78% (19 of 1070; CI 1.46–2.09%) in Lower Bavaria, 4.67% (56 of 1200; CI 4.17–5.17%) in Upper Bavaria, and 11.17% (292 of 2615; CI 10.42–11.91%) in Swabia. In total, 5.51% (375 of 6801; CI 4.97–11.17%) of bovines in Bavaria had detectable levels of BoHV-2 specific antibodies.

The chi-squared test indicated that a statistically significant difference for BoHV-2 seroprevalences on the individual animal level exists when comparing Swabia, Upper Bavaria, and Lower Bavaria (*p* < 0.001) to North Bavaria (Table 1a).

**Table 1:** Results of BoHV-2 serology in relation to the region of origin of animals (a) and farms (b)

***a Individual animal level***				
	**BoHV-2 seropositive animals (*n*** **= 375)**	**BoHV-2 seronegative animals (*n*** **= 6426)**	**OR**^a^**(CI 95%)**	***p*-value**
**North Bavaria**	8	1908	**1.00**	–
**Lower Bavaria**	19	1051	**4.31** (1.88–9.88)	< 0.001
**Upper Bavaria**	56	1144	**11.67** (5.55–24.58)	< 0.001
**Swabia**	292	2323	**29.98** (14.81–60.67)	< 0.001
***b Farm level***				
	**BoHV-2 seropositive farms**^b^**(*n*** **= 78)**	**BoHV-2 seronegative farms**^c^**(*n*** **= 328)**	**OR**^a^**(CI 95%)**	***p*-value**
**North Bavaria**	5	100	**1.25** (0.29–5.40)	0.764462
**Lower Bavaria**	3	75	**1.00**	–
**Upper Bavaria**	17	65	**6.54** (1.83–23.32)	< 0.05
**Swabia**	53	88	**15.06** (4.52–50.15)	< 0.001

^a^Odds Ratios

^b^farms are classified as BoHV-2 seropositive when at least one BoHV-2 seropositive animal is kept

^c^farms are classified as BoHV-2 seronegative when none of the tested animals had antibodies against BoHV-2

Versus Lower Bavaria, the region with the lowest BoHV-2 seroprevalence on the farm level, statistically significant differences for Swabia and Upper Bavaria (*p* < 0.05), but not for North Bavaria (*p* = 0.765) were calculated (Table 1b).

**Fig. 3:**
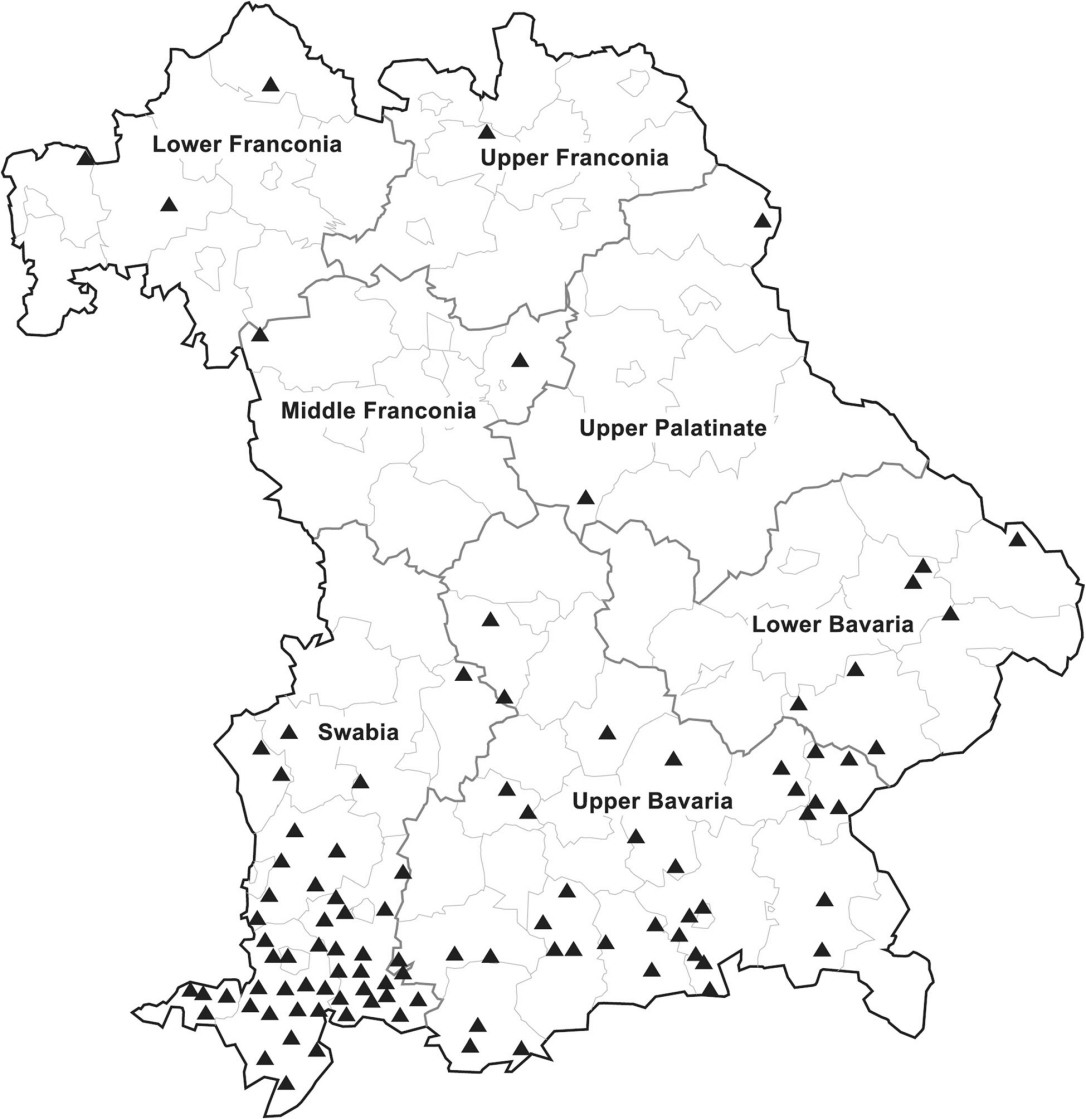
Map of Bavaria indicating the geographical distribution of BoHV-2 seropositive results. The seven administrative regions are shown. Swabia, Upper, and Lower Bavaria represent South Bavaria whereas Upper, Middle, and Lower Franconia as well as Upper Palatinate are addressed as North Bavaria in this study. Black triangles (▲) indicate postal code areas where at least one BoHV-2 antibody positive animal was detected. The data is not quantitative as 561 positive samples were categorized into 100 postal code areas. The map was generated using the software GfK Regiograph Analysis and Adobe Illustrator

The geographic origin of all individual samples that were found to contain BoHV-2 specific ELISA antibodies in the wider context of this study (*n* = 561) is visualized in Fig. 3. In addition to the 375 samples mentioned above relating to the seroprevalence calculations another 186 BoHV-2 seropositive samples were localized. As the latter samples were not randomly selected, the map merely gives a general overview of the geographical distribution across all regions of Bavaria.

### Variability of BoHV-2 antibodies within positive herds

**Fig. 4:**
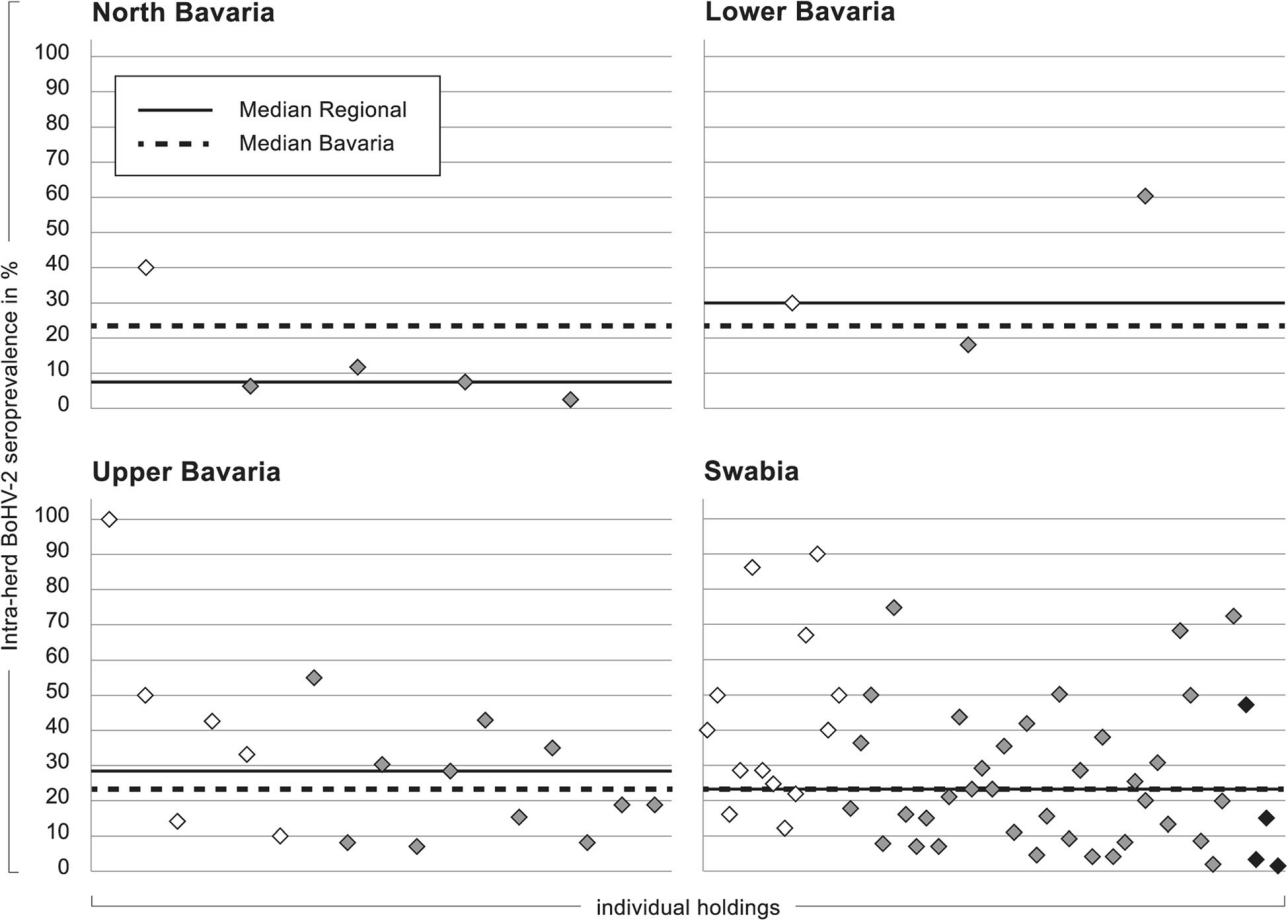
BoHV-2 intra-herd seroprevalences calculated for farms keeping at least one BoHV-2 seropositive animal. BoHV-2 antibodies were determined by ELISA and in case of doubtful or increased reactivities, were confirmed by SNT. One diamond is attributed to one farm. White diamonds (♢) represent farms with one to ten animals tested (*n* = 20), grey ones () farms with eleven to 50 animals tested (*n* = 54), and black ones (♦) farms with more than 50 animals tested (*n* = 4). Solid horizontal lines indicate the median seroprevalence for the respective region. Dotted lines represent the median of intra-herd BoHV-2 seroprevalences calculated for all of Bavaria

In a next step, individual intra-herd BoHV-2 seroprevalences were calculated as illustrated in Fig. 4. Farms in North Bavaria had the lowest prevalence of seropositive animals per farm, with a median of 7.69% in five farms, followed by Swabia (24.26%; 53 farms), Upper Bavaria (28.57%; 17 farms), and Lower Bavaria (30.00%; three farms). However, as the overall number of positive farms markedly varied between regions, these data are difficult to compare. In any case, it was shown that intra-herd seroprevalences are indeed extremely variable, ranging from 2 to 100%.

### Comparison of BoHV-2 seroprevalences with the incidence of inexplicable reactions in the BoHV-1 antibody testing

The interest in BoHV-2 infections and prevalences in Germany has increased after eradicating BoHV-1. One aspect was that BoHV-2 sero-reactivity was related to otherwise inexplicable reactions in the sero-surveillance of BoHV-1 infections, particularly in specific regions of Bavaria [26]. To match the BoHV-2 antibody presence and the respective results in BoHV-1 surveillance, the same 6801 samples were used.

Comparing the results of the group of cattle with inexplicable reactions in the BoHV-1 surveillance with those of the clearly BoHV-1 antibody ELISA negative group, statistically significant differences concerning BoHV-2 prevalences (*p* < 0.001, chi-squared test) were confirmed (Table 2).

**Table 2:** Statistical relation between the results of BoHV-2 serology in relation to the respective results in BoHV-1 ELISAs for animals (a) and farms (b)

***a Individual animal level***				
	**BoHV-2 seropositive animals (n = 375)**	**BoHV-2 seronegative animals (n = 6426)**	**OR**^**a**^**(CI 95%)**	***p*-value**
**BoHV-1 ELISA negative animals**	312	6343	**1.00**	–
**Trachitest-positive animals**	30	60	**10.71** (6.46–15.99)	< 0.001
**Non-negative animals**	33	23	**29.17** (16.92–50.27)	< 0.001
***b Farm level***				
	**BoHV-2 seropositive farms**^**b**^**(n = 78)**	**BoHV-2 seronegative farms**^**c**^**(n = 328)**	**OR**^**a**^**(CI 95%)**	***p*-value**
**BoHV-1 ELISA negative farms**^**d**^	36	278	**1.00**	–
**Trachitest-positive farms**^**e**^	15	37	**3.13** (1.57–6.26)	< 0.001
**Non-negative farms**^**f**^	27	13	**16.04** (7.60–33.86)	< 0.001

^**a**^Odds Ratios

^**b**^farms are classified as BoHV-2 seropositive when at least one BoHV-2 seropositive animal is kept

^**c**^farms are classified as BoHV-2 seronegative when none of the tested animals had antibodies against BoHV-2

^**d**^farms are classified as BoHV-1 ELISA negative when none of the tested sera showed reactivity in the Trachitest

^**e**^farms are classified as Trachitest-positive when at least one animal with reactivity in the Trachitest but none with reactivity in the gB-ELISA is kept

^**f**^farms are classified as non-negative when at least one animal with reactivity in the Trachitest and the gB-ELISA (gE-ELISA negative) is kept

**Fig. 5:**
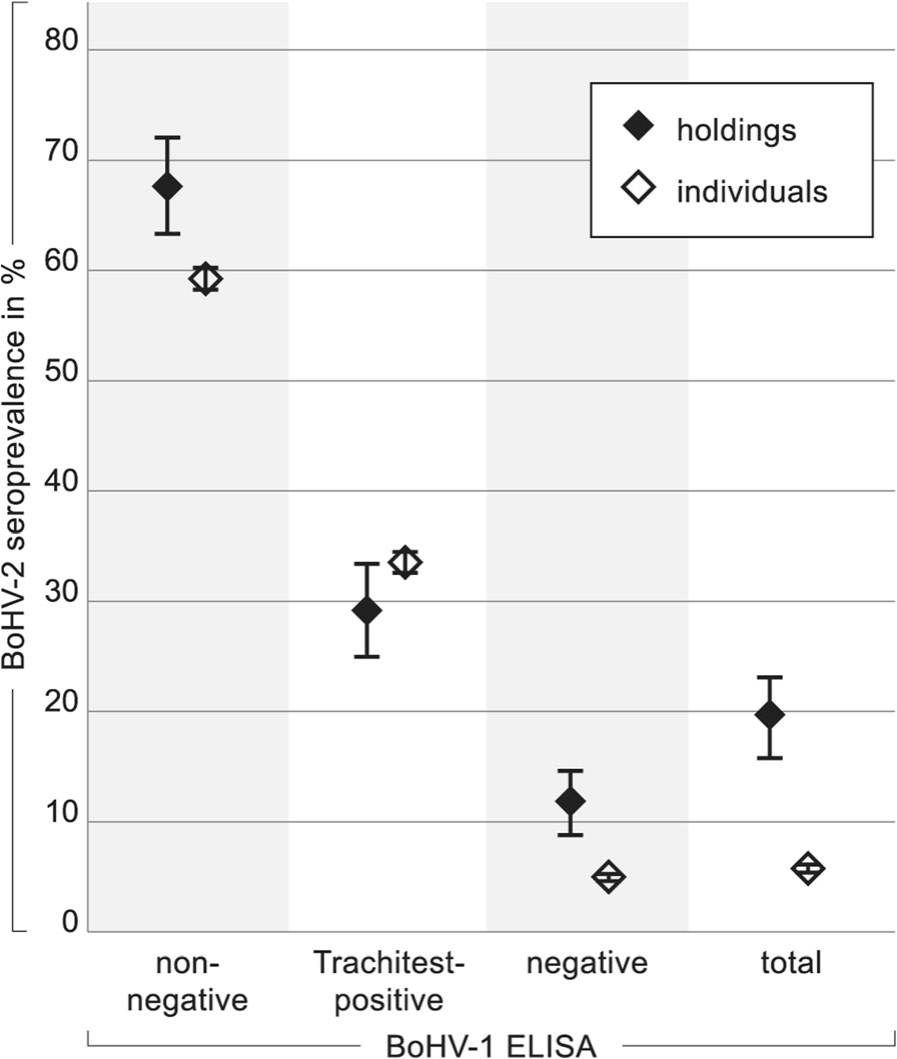
BoHV-2 seroprevalences in relation to BoHV-1 ELISA results. Black diamonds (♦) indicate results when interpreted for holdings, open diamonds (♢) when individual animals are considered. Farms where at least one BoHV-2 seropositive animal is kept are classified “BoHV-2 seropositive”. With respect to results in the BoHV-1 ELISAs, holdings are categorized as follows: “BoHV-1 ELISA negative” (Trachitest negative), “Trachitest-positive” (Trachitest positive but gB-ELISA negative), “non-negative” (Trachitest and gB-ELISA positive or suspect, but gE-ELISA negative). The mean data for 406 holdings and 6801 cattle is given as “total”

Actually, 33.33% (CI 32.21–34.45%) of 90 samples that reacted in only one BoHV-1 ELISA system, the Trachitest, and even 58.93% (CI 57.76–60.10%) of 56 samples with non-negative reactions (= reactions in two BoHV-1 ELISAs), were at the same time BoHV-2 seropositive (Fig. 5). Furthermore, only 4.69% (CI 4.19–5.19%) of the BoHV-1 ELISA negative samples (*n* = 6655) were BoHV-2 seropositive. Taken together, BoHV-1 Trachitest-positive samples had an elevenfold (CI 6.46–15.99) increased probability for BoHV-2 seropositivity compared to BoHV-1 ELISA negative samples (Table 2a). This probability was yet twenty-nine fold increased for samples with non-negative BoHV-1 ELISA results (CI 16.92–50.27).

Reversed, the correlation was not as evident. Of 375 samples in which BoHV-2 antibodies were detected, only 33 reacted non-negative, 30 Trachitest-positive, and the remaining 312 were clearly BoHV-1 ELISA negative. Interpretation of the data on the farm level revealed similar results. 67.5% (CI 62.94–72.06%) of the farms classified as BoHV-1 ELISA non-negative (*n* = 40), 28.85% (CI 24.44–33.25%) of the Trachitest-positive farms (*n* = 52), and 11.46% (CI 8.37–14.56%) of the BoHV-1 ELISA negative farms (*n* = 314) were simultaneously categorized as BoHV-2 seropositive (Fig. 5). Table 2b shows the statistical relation between the results of the BoHV-2 serology and the BoHV-1 ELISAs on the farm level.

### Approaches to detect acute BoHV-2 infections in Bavaria

Another aim of this study was to identify acute BoHV-2 infections. Thus, a BoHV-2 specific real time PCR targeting sequences within the glycoprotein B (gB) gene was designed. Additionally, a published conventional PCR protocol with primers targeting sequences within the BoHV-2 DNA polymerase gene was used. Both were found to detect about 20 copies of BoHV-2 strain “Riems 8/85” (RVB-0064) per reaction.

In a first series of experiments, 193 tissue samples and 918 nasal swabs were analyzed by BoHV-2 specific real time PCR. A slight rise in fluorescence was observed during the final cycles for several tissue, as well as swab samples, therefore these samples were retested by the BoHV-2 DNA polymerase PCR. However, in no case a specific amplification of BoHV-2 sequences resulted.

The PCR protocols used in this study base on the sparse sequence information available, and, on top of that, from older or non-European BoHV-2 isolates. To nevertheless ensure that BoHV-2 sequences were not missed, an even broader PCR-format, a PanHerpes PCR, was used hereafter [33]. All 193 tissue samples mentioned above as well as 46 additional tissue samples were tested applying this protocol (see methods). Herpesvirus DNA was specifically amplified out of 40 samples, stemming from 25 different animals (24 bovines and 1 goat). Unfortunately, nucleic acid sequence determination (Sanger Sequencing, Eurofins Genomics) did still not reveal any indication for BoHV-2 specific sequences.

Primarily Gammaherpesviruses were identified. In one case, showing pronounced mucosal lesions, a bovine OvHV-2 *(Ovine gammaherpesvirus 2;* family *Herpesviridae*, subfamily *Gammaherpesvirinae*, genus *Macavirus)* infection was diagnosed. Furthermore, in the udder lymph node of a goat, sequences of the *Caprine gammaherpesvirus 2* (CpHV-2; family *Herpesviridae*, subfamily *Gammaherpesvirinae*, genus *Macavirus*) were detected. Remaining sequences will be assessed in more detail in future studies.

Taken together, although a broad spectrum of samples and types of lesions were included in this study, so far, no BoHV-2 genomes were detected. In principle, the suitability of the strategy applied, however, was proven by the amplification of several herpesvirus sequences – other than BoHV-2.

## Discussion

The aim of this study was to improve the knowledge about the epidemiology and virology of BoHV-2 infections. For the analysis of 6801 bovine blood sera the commercially available indirect BoHV-2 ELISA ID Screen® BHV-2 Indirect (ID VET) was used. Considering doubtful results as positive (*n* = 20), 6552 out of 6801 sera could be classified as clearly positive or negative. However, it was of interest to sensitively detect even low antibody levels, as previous reports had indicated that BoHV-2 specific antibodies might not persist for long if reactivation doesn’t occur [8]. Therefore, the SNT was additionally consulted for the rating of samples with increased ELISA results (50% ≤ S/P% ≤ 90%; Fig. 1). Yet, the seroprevalences determined might still be slightly underestimated, assuming the existence of individual, low level antibody positive sera within the pool of sera with results below the cut-off of 50%. The effect on the discussed results would, however, be minor.

Overall, 5.51% of the sera tested throughout Bavaria contained antibodies against BoHV-2 and a marked South-North divide was demonstrated (Figs. 2 and 3). Interestingly, in the late 70s a regional distribution in Switzerland was discussed with BoHV-2 antibody ratios ranging from 1 to 19% [24]. The authors speculated that BoHV-2 infections might actually be spreading from Italy and the South to the North and West. In the same way one could either argue that BoHV-2 infections in Bavaria are actually slowly spreading towards the North, or, that other regional issues exist which might locally favor BoHV-2 infections. If spreading actually occurs, it could be hypothesized on the one hand that BoHV-2 was to some extend “co-eliminated” in the context of BoHV-1 eradication and is now slowly reintroduced into Bavaria. On the other hand, BoHV-2 infections per se could be - for other reasons - a relatively new or statistically still minor phenomenon for Bavaria. BoHV-1 freedom could then even favor BoHV-2 expansion with time. Other hypotheses could argue toward a local phenomenon. Firstly, only one third of the Bavarian cattle population is located in North Bavaria. The regions with the highest BoHV-2 seroprevalences, Swabia and Upper Bavaria, are also the regions with the largest cattle population (about 50% of the Bavarian cattle population), which might facilitate virus transmission between herds.

Secondly, and even more importantly, whereas in North Bavaria cattle are mostly kept in stables, pasture management is common in the South. Moreover, in areas close to the Alps traditional alpine farming is practiced. An increased contact of cattle to people, to cattle from other herds, and also to other domestic or wild animals results. Wild ruminants like red deer, roe deer, fallow deer, mouflon, and chamois are also susceptible for BoHV-2 [18, 34]. It is not known if these species develop clinical symptoms of BHM, but a function as reservoir hosts might be possible.

Finally, cattle on pastures, especially on alpine pastures, are exposed to more extreme climate conditions than cattle in stables. Factors like UV radiation or marked temperature fluctuations might favor reactivation of latent infections and thus virus spread and/or the re-stimulation of antibody responses. The effect of skin temperature on BoHV-2 infections has been clearly shown previously, proposing an explanation for the seasonality of BHM-outbreaks [8, 35].

Interestingly, not only the overall seroprevalence, but also the intra-herd prevalence was altogether relatively low in this study, but at the same time very variable, ranging from 2 to 100% (Fig. 4). In some herds, only one seropositive animal was found. In contrast, early case reports describe up to 96% of the cows within a herd being clinically affected [2]. Obviously, transmission of current and possibly local BoHV-2 strains, under actual husbandry conditions in Germany, is by far not as effective as formerly described.

This conclusion was somewhat corroborated by the results of the BoHV-2 genome analyses in this study. To get an initial idea about BoHV-2 infections in the region, various samples taken from 111 animals with lesions such as skin alterations at udder, teats, muzzle, or mouth were analyzed as well as 918 nasal swabs. By choosing these materials, acute as well as reactivated or latent infections were targeted. For BoHV-2, which is a member of the subfamily *Alphaherpesvirinae*, it is reasonable to assume that seropositive animals are virus carriers, and reactivation experiments have proven this concept in the past [10, 12, 36]. Therefore, the working hypothesis was that in the respective regions about 5% (Upper Bavaria) to 11% (Swabia) of the animals should be either in a state of acute or latent infection. To sensitively detect any status of infection PCR was the method of choice. Various protocols targeting several regions of the BoHV-2 genome that should be highly conserved were used. In addition, including a PanHerpes approach for all tissue samples should have ensured that BoHV-2 genomes – if present – would not have been missed. If genomes had been detected, in a next step virus isolation would have been attempted for those samples indicating an early stage of productive infection. However, none of the clinical lesions analyzed was caused by an acute or reactivated BoHV-2 infection. This leads to the conclusion that at the moment BoHV-2 clinical disease might not be considered a severe problem. This is supported by the fact that neither veterinarians in the field have sent in BoHV-2 positive samples – although repeatedly invited - nor pathologists in our pathology department were able to sample acute or reactivated infections. The latter had been hypothesized to putatively occur as a side effect of the immunosuppression caused by others diseases.

Virus isolation from nasal swabs upon experimental infection and reactivation has been reported [11]. However, specific DNA sequences could not be detected in swabs analyzed in this study. This might indicate that reactivation and shedding via the respiratory tract is not a common event in the field.

Taken together, it can be assumed that the types of test materials as well as the detection methods, which were used for this study, would be appropriate to find BoHV-2 genomes if present in detectable amounts. It is nevertheless possible that an even higher degree of awareness for corresponding, possibly even discrete and mild clinical signs in the field, would help to monitor the disease and putative future changes in epidemiology.

From the administrative point of view, specifically aiming at a smooth sero-surveillance of BoHV-1 infections in an article 10-region, the further evolution of BoHV-2 infections needs to be kept in view. According to the current legal basis, only gE-ELISA positive sera are indicative for an infection with BoHV-1 wild type virus [37]. Competitive gE-ELISAs are highly specific and were developed originally to allow for the differentiation between field virus infected and vaccinated individuals (DIVA). This principle was essential during intermediate stages in the German BoHV-1 eradication strategy. However, now, several years after the complete region of Bavaria has been declared free of BoHV-1 infections, specific antibodies should not be present anymore. Nevertheless, now and again single samples turn up with conspicuous results in BoHV-1 specific ELISAs, with the exception of the gE-ELISA. There is no epidemiological foundation to explain these “non-negative” reactions. Early field virus infections, when antibodies against gE might not yet be detectable, are consistently ruled out by retesting after a reasonable time interval. Furthermore, vaccination has been banned throughout Bavaria in 2011. Remaining vaccinated animals should thus only provide a very minor and documented population.

A currently completely unexplained, but statistically significant correlation between the BoHV-2 seroprevalence and these reactions in the BoHV-1 surveillance system within a herd was corroborated in this study (Fig. 5). About 60% of samples from cattle with inexplicable, non-negative reactions in the BoHV-1 Trachitest and gB-ELISA, but not the gE-ELISA, were at the same time BoHV-2 seropositive. On the farm level, nearly 70% of sampled farms with single non-negative BoHV-1 reactivities among their cattle were also classified as BoHV-2 seropositive. Interestingly, not necessarily the same individuals were affected. In fact, 40% of samples from cattle with non-negative reactions were clearly negative for BoHV-2 antibodies and 5% of the cattle population classified as BoHV-1 negative was nevertheless BoHV-2-seropositive. In any way, direct cross-reaction of BoHV-2 specific sera with BoHV-1 antigen can be excluded, as has been repeatedly shown [26, 28, 29]. Furthermore, initial experiments of the current study revealed no cross-neutralization in BoHV-2 specific and BoHV-1 specific SNTs. Therefore, a reason for these observations can only be estimated at this point. A BoHV-2 infection could for example influence the BoHV-1 ELISA sero-reactivity under specific circumstances only, like after several reactivations or in combination with a third, as yet unidentified infection or factor. Furthermore, BoHV-2 infection and developing non-negative BoHV-1 test results might not even be directly connected, but could both be independently linked to a common regional risk or husbandry condition. Further studies will be necessary to assess these hypotheses.

However, the difference between the current, somewhat broader, regional distribution of non-negative BoHV-1 ELISA results, when compared to the ones discussed by Böttcher, Boje [26], might indicate a further dissemination over time. Before, it was reported that the non-negative problematic is specific for Swabia [26]. It is possible that, as nearly ten years have passed between the sample takings, BoHV-2 infections – potentially concurrent with non-negative results in BoHV-1 ELISAs - have spread from Swabia to Upper Bavaria. Anyhow, as already discussed above, a comparison of the results of the two studies is not valid, as aims and study designs differed too much.

Taken together, both issues – BoHV-2 infections and the occurrence of non-negative reactions in BoHV-1 surveillance – are still extremely rare in the North, but the apparently relative fast developments in the Southern regions show how important a close surveillance of BoHV-2 infections in the following years after BoHV-1 eradication will be.

Last but not least, very sporadically in sera, that appeared “non-negative” and that were tested for neutralizing antibodies against BoHV-1 and BoHV-2 in the context of this study, BoHV-1 neutralization was detected. Follow up investigations always allowed to trace these antibodies back to an earlier vaccination. More importantly though, taking these results into account together with the observation that a one to one cross-reactivity between BoHV-1 and BoHV-2 positive sera in the ELISAs does not exist, the conclusion that a non-negative reaction can easily be explained by a BoHV-2 infection of the respective animal cannot be drawn without further consideration.

## Conclusions

In summary, BoHV-2 specific antibodies were diagnosed in samples all over Bavaria and statistically valid seroprevalences could be presented for the first time. Interestingly, significant regional differences in BoHV-2 seroprevalences were discovered. The highest prevalence, when looking at individual animals, was detected for Swabia, in the South-West, followed by Upper Bavaria, Lower Bavaria, and finally North Bavaria. Moreover, the intra-herd prevalence was shown to vary markedly, indicating that tendency for spreading within a holding is not yet predictable and, more importantly, that more information on the epidemiology and virology of BoHV-2 is severely needed. In addition, a previously discussed statistical correlation between problems in the BoHV-1 surveillance and BoHV-2 sero-reactivity [26] was corroborated in this study. However, as expected for viruses that are genetically and antigenically not too closely related, no direct cross-reactivity of sera in BoHV-1 or BoHV-2 specific antibody tests were found. Furthermore, attempts to directly identify BoHV-2 infections have failed so far, since BoHV-2 genomes could not be detected, neither in any of the 239 selected tissue samples nor in any of the 918 clinical nasal swabs.

Taken together, these findings indicate that BoHV-2 infections currently do not present a major clinical problem in Bavaria. Nevertheless, considering the demonstrated statistical correlation with inexplicable reactions in the BoHV-1 surveillance and the indications for further spreading, BoHV-2 infections need to be further monitored and more data is required to understand these current epidemiological issues.

## Methods

### Serological investigation

#### Samples and statistical methods

The aim of the study was to determine regional BoHV-2 seroprevalences with a statistical certainty of 95% and an accuracy of at least 5%. According to the data provided by Böttcher, Boje [26], it was assumed that Swabia was more affected than other administrative regions of Bavaria. For this reason, a putative prevalence of about 10% for Swabia and 5% for Upper Bavaria, Lower Bavaria, and North Bavaria (Upper Palatinate, Upper, Middle, and Lower Franconina) and infinite cattle populations were taken as a basis in a first calculation step. Sample sizes of at least 139 farms from Swabia and 73 farms from the other regions resulted [38]. In a second step, the distribution of the farms to be selected was adapted to the respective regional cattle densities.

Out of the large pool of blood samples from healthy animals that were sent in in 2017, 2018, and in the beginning of 2019 to the Bavarian Health and Food Safety Authority, 6801 sera stemming from 406 farms were randomly chosen and included in this study. Following the sampling scheme explained above, finally 141 farms (2615 sera) from Swabia, 82 farms (1200 sera) from Upper Bavaria, 78 farms (1070 sera) from Lower Bavaria, and in summary 105 farms (1916 sera) from the four administrative regions of North Bavaria, i.e. Upper Palatinate, Upper Franconia, Middle Franconia, and Lower Franconia, were included.

In order to better describe seroprevalences, confidence intervals (CI) that should cover the true seroprevalence with a certainty of 95%, were used. To check for the relationship between BoHV-2 seroprevalence and sample origin on the one hand and between BoHV-2 seroprevalence and BoHV-1 test results on the other hand, the chi-squared test was applied. A quantification of these relationships was made by odds ratios. Values of *p* < 0.05 were regarded as statistically significant.

#### ELISAs detecting BoHV-1 or BoHV-2 antibodies

To detect antibodies against BoHV-2 an indirect ELISA (ID Screen® BHV-2 Indirect; ID VET) was used. A purified French BoHV-2 isolate serves as antigen [39]. According to the manufacturer’s instructions, sera are classified as positive, when the sample to positive control-ratio (S/P%) is greater or equal 110%, as negative, when the S/P% is less or equal 90%, and as doubtful, when the S/P% lies in between (90% < S/P% < 110%). Using these cut-off values, a diagnostic sensitivity of 92.0% or 96.6% and a respective diagnostic specificity at 100.0% or 98.2% has been determined depending on whether or not doubtful results are considered positive (ID VET: Internal validation report ID Screen® BHV-2 Indirect; available upon request). The underlying data largely bases on the results of a study analyzing a large number (*n* = 424) of preselected samples in the ELISA and the BoHV-2 specific serum neutralization test (SNT) as a gold standard [40]. To increase the sensitivity in this study an additional range of sample/positive control ratios (S/P%) was considered. Ratios between 50 and 90%, hence lower still than the test systems range of “doubtful” results (90% < S/P% < 110%), were defined as “increased”. Samples with “doubtful” results (*n* = 20) were retested by BoHV-2 specific SNT. “Increased” results (*n* = 62) were controlled accordingly for all sera that were suitable for this test system (*n* = 61). One sample had to be excluded as it caused toxic reactions in cell culture. Furthermore, to ensure the comparability of these low level antibody results between ELISA tests, an additional weak positive reference serum (“MRI-BHV-2”, BoHV-2 positive, freeze dried serum, IDVET) was used in every assay.

In Germany, a variety of commercially available ELISA test kits are registered for the official testing of cattle sera for antibodies against BoHV-1. Three different BoHV-1 antibody ELISA systems were applied in a hierarchical order: To begin with, an indirect virus ELISA, hereafter called Trachitest (IDEXX Trachitest Serum Screening, IDEXX Europe B.V.), was used. According to the manufacturer’s instructions samples have to be retested when reactivity exceeds 35% but does not yet reach positive values (≥45%). However, to increase the sensitivity within the framework of the present study, this cut-off value was reduced to 25%. In case of a reactivity above this value the test was repeated and additionally samples were analyzed in a second ELISA, a competitive gB-ELISA (IDEXX IBR gB X3, IDEXX Europe B.V. or cattletype BHV1 gB Ab, Indical). In case of positive or suspect results in the gB-ELISA, according to the test specifications, the third ELISA, a competitive gE-ELISA (IDEXX IBR gE, IDEXX Europe B.V.) was used. Finally, considering the results of the three ELISAs, sera were categorized using the following nomenclature: “BoHV-1 ELISA negative” (Trachitest negative), “Trachitest-positive” (Trachitest positive, gB- ELISA negative), “non-negative” (Trachitest and gB-ELISA positive or suspect, gE-ELISA negative), and “BoHV-1 antibody positive” (all assays, especially gE-ELISA, positive). The latter definition is in accordance with German BoHV-1 regulations, determining procedures to protect cattle against BoHV-1 infections [37].

#### Serum neutralization test (SNT)

BoHV-1-strain “Schönböken” (RVB-0073) and BoHV-2-strain “Riems 8/85” (RVB-0064), both obtained from the Collection of Viruses in Veterinary Medicine, Friedrich-Loeffler-Institute, Insel Riems, Germany, were propagated on KOP-R cells, a diploid bovine esophageal cell line (RIE244, Collection of Cell Lines in Veterinary Medicine (CCLV), Friedrich-Loeffler-Institute, Insel Riems, Germany). Standard procedures were followed. Briefly, KOP-R cells were cultivated for about 24 h in 96-well tissue culture plates at 37 °C/5% CO_2_ in MEM with Earle’s salts and L-glutamine (Gibco™ by life technologies, USA) supplemented by 10% gamma irradiated fetal bovine serum (FBS; Biowest, South America), 1% non-essential amino acids and 1% pyruvate.

Heat-inactivated blood sera (30 min, 56 °C) were diluted in log2-steps and incubated with 100 TCID_50_ of the respective virus in a 1 + 1 ratio at 37 °C and 5% CO_2_ overnight. The following day the pre-incubated serum-virus-mixtures were transferred to microtiter plates containing KOP-R cells in monolayers at a confluence of about 70–80%. At this point, the FBS content had been reduced to 2%. After incubating at 37 °C/ 5% CO_2_ for another 72 h, cells were evaluated for virus growth using an inverted microscope. A replicate was considered infected when at least one focus was observed. Vice versa neutralization of infectivity was scored only when neutralization was complete and no cytopathic effect at all could be observed.

### PCR-analyses

#### Samples

Two hundred thirty-nine tissue samples from teat-, udder-, or muzzle lesions (*n* = 113), corresponding lymph nodes (*n* = 85), and in some cases trigeminal ganglia (*n* = 41) from altogether 111 animals (107 bovines, 3 sheep, 1 goat) were included in this study. As BoHV-2 antibodies were clearly more prevalent in South Bavaria, the sampling focused on this region. Practitioners in the field were asked for special attention to udder and teat lesions and to send in samples. Most samples, however, were taken at the pathology department of the Bavarian Health and Food Safety Authority. It was decided to test tissues from all kinds of visible lesions at the udder and the teats, to ensure that no putative disease status – including reactivation – was missed.

Furthermore, lesions at the muzzle and mucosa of the mouth of calves had been reported in the context of BoHV-2 infections, as well as virus isolation not only from these lesions but also from nasal swabs during acute infection and upon experimental reactivation [11]. In consequence, not only such lesions were taken as an indication for analysis, but also case histories of respiratory disease or mucosal reddening. Respective nasal swabs (*n* = 918) were included with the intention to possibly detect reactivated BoHV-2 infections in the course of stress and clinical disease of any other cause.

#### PCR

Nucleic acids from tissue samples were either extracted using a magnetic processor (BioSprint96, Qiagen) and the BioSprint96 One-For-All Vet Kit or manually with the QIAamp DNA Mini Kit (Qiagen). Nucleic acids from nasal swabs were purified by means of the QIAamp Viral RNA Mini Kit (Qiagen), which has been thoroughly validated and is routinely used also for the detection of viral DNA in the laboratory. As to date very little sequence information is available on BoHV-2 genomes and as the aim of the study was the detection of putative actual and local strains in Bavaria, a multi PCR-strategy was chosen.

##### Real time PCR targeting sequences within the glycoprotein B (gB) gene

Published and self-generated sequences of the conserved glycoprotein B gene of different BoHV-2 strains (RVB-0062 and RVB-0064; Collection of Viruses in Veterinary Medicine, Friedrich Loeffler-Institute, Insel Riems, Germany), were used for sequence alignment (Clustal W version 2.1). Two primer and probe combinations, the BoHV2-Mix2.1 qPCR and the BoHV2-Mix2.3 qPCR were applied in parallel. In a total reaction volume of 20 μl (including 5 μl of template DNA) the respective primers (*BoHV2-Mix2.1 qPCR:* BoHV2-gB-2.1F: 5′-GAG GGC ATC GCC GTA ATC-3′, BoHV2-gB-2.1R: 5′- CAG TCA CGG CCT TGT AGT AC-3′; *BoHV2-Mix2.3 qPCR:* BoHV2-gB-2.2F: 5′-CAT CGC CGT AAT CTT CAA GGA-3′; BoHV2-gB-2.1R: see above) were added to a final concentration of 0.8 μM and the probe to 0.2 μM (BoHV2-gB-2FAM: 5′-FAM-ACC TCG CGC CGT ACA AGT TTA AGG C-BHQ1–3′). In a Bio-Rad CFX 96™ Real-Time PCR Detection System DNA was amplified basing on the cycling protocol of the PerfeCta MultiPlex qPCR ToughMix (Quanta BioScience). Amplification started with an initial denaturing step of 10 min (95 °C), followed by 40 cycles of denaturation (95 °C, 15 s) and primer annealing and extension (60 °C, 60s).

##### Conventional PCR targeting sequences within the DNA polymerase gene

The protocol described by Cargnelutti, Weiblen [41] was slightly altered, in as much as the HotStarTaq Polymerase (Qiagen) was used in a total volume of 50 μl, containing 5 μl template DNA and in addition Q-Solution and MgCl_2_. The cycling protocol differed from the manufacturer’s instructions as annealing was done at 46 °C (60s), as 45 cycles were used, and as the final extension was shortened to 7 min.

##### PanHerpes PCR targeting the highly conserved sequences within the herpesviral DNA polymerase gene

This PCR protocol has been designed and proven valuable for the detection of a broad range of herpesviral genomes [33]. The HotStarTaq polymerase (Qiagen) was used as described above, otherwise following the procedures published by Ehlers et [33].

For the newly established real time PCR and the conventional PCRs targeting the DNA-polymerase or the gB gene, specificity was tested using the following putatively contaminating viruses: BoHV-1, *Equid alphaherpesvirus 1* (family *Herpesviridae*, subfamily *Alphaherpesvirinae*, genus *Varicellovirus*), *Bovine gammaherpesvirus 4* (family *Herpesviridae*, subfamily *Gammaherpesvirinae*, genus *Rhadinovirus*), OvHV-2), *Orf virus* (family *Poxviridae*, subfamily *Chordopoxvirinae*, genus *Parapoxvirus*), *Pseudocowpox virus*, (family *Poxviridae*, subfamily *Chordopoxvirinae*, genus *Parapoxvirus*), *Bovine papular stomatitis virus* (family *Poxviridae*, subfamily *Chordopoxvirinae*, genus *Parapoxvirus*), *Lumpy skin disease virus* (family *Poxviridae*, subfamily *Chordopoxvirinae*, genus *Capripoxvirus*), *Bluetongue virus-4* and *-8* (family *Reoviridae*, subfamily *Sedoreovirinae*, genus *Orbivirus*), *Pestivirus* (formerly: Bovine viral diarrhea virus; family *Flaviviridae*, genus *Pestivirus*), and samples containing virus of the genus *Orthopoxvirus* (family *Poxviridae*, subfamily *Chordopoxvirinae*) or of the family *Papillomaviridae*. PCR protocols were also analyzed for reproducibility and sensitivity. Sensitivity was calculated in numbers of genome copies that were reproducibly detectable. The efficiency of nucleic acid purification as well as the influence of inhibitory effects was controlled for all samples detecting beta-actin sequences by real time PCR as it has been described [42].

## Data Availability

The datasets used and analysed during the current study are de-identified and available from the corresponding author on reasonable request.

## Abbreviations

BHM: Bovine herpes mammillitis
BoHV-1/-2: Bovine Alphaherpesvirus 1/2
CI: Confidence interval
CpHV-2: Caprine Gammaherpesvirus 2
DIVA: Differentiation of infected and vaccinated animals
ELISA: Enzyme-linked immunosorbent assay
FBS: Fetal bovine serum
gB/E: Glycoprotein B/E
KOP-R: Bovine oropharyngeal cells
MEM: Minimum essential medium
OR: Odds Ratios
OvHV-2: Ovine Gammaherpesvirus 2
PCR: Polymerase chain reaction
SNT: Serum neutralization test
